# Role of Matrix Metalloproteinase-8 in Atherosclerosis

**DOI:** 10.1155/2013/659282

**Published:** 2013-01-09

**Authors:** Sébastien Lenglet, François Mach, Fabrizio Montecucco

**Affiliations:** ^1^Cardiology Division, Department of Medicine, Geneva University Hospital, Foundation for Medical Research, 1211 Geneva 4, Switzerland; ^2^Clinic of Internal Medicine 1, Department of Internal Medicine, University of Genoa, 16100 Genoa, Italy

## Abstract

Plaque rupture is the main cause of acute myocardial infarction and stroke. Atherosclerotic plaques have been described to be vulnerable and more prone to rupture when they are characterized by thin, highly inflamed, and collagen-poor fibrous caps and contain elevated levels of proteases, including metalloproteinases (MMPs). Initiation of collagen breakdown in plaques requires interstitial collagenases, a MMP subfamily consisting of MMP-1, MMP-8, and MMP-13. Previous reports demonstrated that MMP-1 and MMP-13 might be overexpressed in both human and experimental atherosclerosis. Since neutrophils have been only recently reported in atherosclerotic plaques, the role of MMP-8 (formerly known as “neutrophil collagenase”) was only marginally evaluated. In this paper, we will update and comment on evidence of the most relevant regulatory pathways and activities mediated by MMP-8 in atherogenesis.

## 1. Introduction

To date, the MMP family comprises 25 structurally and functionally related members, of which 24 are found in mammals. All MMPs are characterized by a shared multidomain structure and, in particular, a highly conserved catalytic domain consisting of a Zn^2+^-binding consensus sequence. Based on their primary structure and substrate specificity, MMPs can be classified into five groups: collagenases (MMP-1, -8, and -13), gelatinases (MMP-2 and -9), stromelysins (MMP-3, -10, and -11), minimal MMPs (MMP-7 and -26) and membrane-type MMPs (MT-MMPs). Most MMPs are synthesized and secreted as inactive proenzymes or zymogens (pro-MMP) and require a proteolytic process to become active. Given their relevant “cannibal” potential of MMPs, their biological activity is tightly controlled. This regulation may take place at the levels of gene transcription, translation, and proenzyme activation. Finally, the activity of MMPs might be regulated by other inhibitory proteins, such as *α*2-macroglobulin (a plasma protein that acts as a general proteinase inhibitor) and specific tissue inhibitors of MMPs (TIMP-1, -2, -3, and -4). The biochemical properties and local expression patterns have been shown to actively determine the net resultant MMP activity within tissues, mainly characterized by the balance between the levels of activated MMPs and TIMPs [1].

Together with other collagenolytic enzymes (such as the cysteine proteases cathepsins K and L) [2, 3], MMPs have been shown to play a critical role in inflammatory processes underlying plaque rupture. Several studies have demonstrated that MMP overexpression was positively associated with the destruction of the extracellular matrix (ECM) at the vulnerable shoulders of human atheroma [4]. For example, Sukhova and coworkers have shown that the increased collagenolysis in atheromatous plaques is mediated by the interstitial collagenases MMP-1 and MMP-13 [5]. Indeed, these proteases are able to cleave the triple helix of collagens. Another promising candidate widely investigated in observational studies in atherosclerosis is represented by MMP-9. Effectively, the upregulation of intraplaque MMP-9 leads to the increase of plaque hemorrhage and rupture in mouse models [6]. The analysis of human coronary lesions has revealed active synthesis of MMP-9 by macrophages and smooth muscle cells (SMCs) in plaques of patients with acute coronary syndromes but not in those with stable angina [7]. Also neutrophils have been shown to colocalize with MMP-9 in mouse roots plaques, suggesting these cells to be as a potential source of intraplaque MMP-9 [8]. Furthermore, peripheral serum levels of MMP-2 and -9 have been shown to be increased in patients with acute coronary syndromes, suggesting an active role in plaque destabilization [9, 10]. However, type I and type II collagens, which account for the load-bearing strength of the plaque cap, are not substrates for the MMP-9 enzyme [11]. In some reports, it has been shown that high concentrations of others gelatinases like MMP-2 can degrade type I collagen in an *in vitro* environment devoided of TIMPs. Nonetheless, it is likely that only *in vivo* the interstitial collagenases are able of degrading these fibrillar collagens and therefore must play a role in the pathogenesis of plaque rupture.

The majority of the studies provided persuasive evidence that gelatinases are critical degrading agents of protective histological structures of the atheroma. The aim of this paper is to update evidence for a direct involvement of the neutrophilic collagenase MMP-8 in atherogenesis and plaque vulnerability.

## 2. Role of MMP-8 in Atherosclerotic Plaque Pathophysiology

Like most MMPs, MMP-8 is secreted as an inactive proenzyme that needs to be activated before it can exert its function. MMP-8 activation can be mediated by reactive oxygen species (ROS) released from activated neutrophils or a variety of proteases like cathepsin G, chymotrypsin, or MMPs (-3, -7, -10, and -14). This suggests that MMP-8 activation is indeed strongly regulated and mostly limited to sites of inflammation than in the systemic circulation. Once activated, MMP-8 can cleave a wide range of substrates. In particular, MMP-8 degrades type I collagen, which is a major component of the fibrous cap (which protects the vessel from rupture and maintains the integrity of the atherosclerotic vessel wall), 3-fold more potently than MMP-1 and -13 [12, 13]. Degradation occurs by cleavage of the collagen molecule at the distinct Gly(775)-Ile(776) position, which generates 2 fragments that are further degraded by nonspecific proteases.

One of the most intriguing MMPs, MMP-8, also known as collagenase-2 or neutrophil collagenase, was long thought to be expressed solely in neutrophil precursors since it has been cloned from RNA extracted from peripheral blood leukocytes of a patient with chronic granulocytic leukemia [14]. In 2001, Herman and coworkers demonstrated that MMP-8 was also expressed by endothelial cells (ECs), smooth muscle cells (SMCs), and macrophages (M*∅*) within human atherosclerotic lesions, thus attesting that MMP-8 expression extends beyond a single-cell type [15]. Indeed, the authors demonstrate that MMP-8 synthesis and release by EC, SMC, and M*∅* require a prolonged exposure to inflammatory cytokines (such as IL-*β* or CD40L), whereas neutrophils store MMP-8 zymogen in intracellular granules and releases the collagenase almost immediately on stimulation. Western blot analysis also revealed that the 55-kDa form of MMP-8 in atheroma corresponds to the active form of the enzyme. Moreover, colocalization experiments demonstrated that MMP-8 is expressed with cleaved type I collagen rather than intact type I collagen. These data strongly support an active proatherosclerotic role of MMP-8 in atherogenesis. 

Shortly after, Dollery and colleagues confirmed the presence of the “neutrophil elastase” in M*∅* of the atherosclerotic plaques [16]. But more accurately, they demonstrated that the robust staining for MMP-8 and M*∅* was localized to the lesion's shoulder, a region prone to rupture, and provokes acute coronary syndromes. On the contrary, the fibrous plaques which possess abundant SMC did not contain immunoreactive MMP-8 as endothelial cells. Biochemical studies corroborated the immunohistochemical findings of increased MMP-8 in atheroma and particularly those with characteristics of vulnerability to rupture. Thus, MMP-8 was 8-fold greater in atheromatous plaques and 3-fold greater in fibrous plaques than in normal vessels. From this study, it appears that the collagenase MMP-8 identified and produced within the plaque may play an important role in the arterial remodeling or in the promotion in plaque rupture. Indeed, MMP-8 has been shown to activate other MMPs (i.e., MMP-2, -3, and -9) and inactivate TIMP-1 [17–19]. Nevertheless, the potential contribution of one of the major sources of MMP-8 (i.e., neutrophils) to atherosclerotic plaque maturation should be also considered in the different phases of atherogenesis. 

Indeed *ex vivo* studies on coronary artery specimens confirmed an increased number of neutrophils in ruptured plaques as compared to intact plaques and suggest that neutrophil infiltration is actively associated with acute coronary events [20, 21]. To infirm this hypothesis, Dorweiler and coworkers have proposed a very elegant model of human neointima formation to study the molecular mechanism underlying human polymorphonuclear phagocytes (PMN) recruitment within atherosclerotic lesions [22]. Authors demonstrated that LDL-induced secretion of IL8 by intimal SMC induced the adherence, transmigration, and local infiltration of PMN into intima. Moreover, the authors showed that the infiltrating PMN released MMP-8 in response to LDL and favored endothelial cell (EC) apoptosis in this experimental setting. Taken together, these results demonstrated that the release of MMP-8 and the subsequent apoptosis of ECs may be linked to fibrous cap thinning and plaque rupture, thus promoting the transformation of stable into unstable lesions in clinical cardiovascular disease [21]

Additional evidence for MMP-8 in plaque rupture has been proposed by Molloy and coworkers [23]. In a cohort of 159 patients, authors showed that the concentrations of the active form of the MMP-8 collagenase were significantly increased in clinically defined unstable carotid plaques. Moreover, MMP-8 protein colocalized with M*∅*-positive areas, thus confirming that these cells are a potential intraplaque source of MMP-8. This study also suggests that the active form of the enzyme is most important in modulating plaque rupture and confirms the pivotal role of macrophage infiltration in plaque destabilization. Since no relevant changes in two collagenolytic enzymes MMP-1 and -13 were observed, these studies further supported a leading role of MMP-8 in the plaque rupture [24, 25]. Analog results with MMP-8 were found in carotid atherosclerotic plaques from a cohort of 150 patients, but the authors also demonstrate increase activity levels for MMP-9 in unstable plaques and MMP-2 in stable lesions, respectively, confirming that the MMPs activities differ among carotid plaque phenotype [26]. Finally, in a cohort of 543 patients undergoing carotid endarterectomy, Peeters and coworkers have shown that, in contrast with MMP-2, increased carotid plaque MMP-8 and MMP-9 levels are associated with an unstable plaque phenotype. Moreover, an increased plaque MMP-8 level was associated with an increased risk for the occurrence of secondary manifestations of atherosclerotic disease during followup [27]. This result hypothesized that the local MMP-8 plaque level might predict atherosclerotic cardiovascular events.

The major breakthrough comes from the study of Laxton and coworkers. They generated ApoE^−/−^ MMP-8^−/−^ mice to support a pathogenic role for MMP-8 in the development and progression of atherosclerosis. MMP-8 knocking out substantially reduced the atherosclerotic extent in Western diet-fed ApoE^−/−^ mice. The atherosclerotic lesions in these mice had fewer macrophages and higher collagen content, suggesting that these modifications could have implications on atherosclerotic lesion stability [28]. MMP-8 possesses also a proteolytic activity on some nonmatrix proteins such as angiotensin I (Ang I) [29]. *In vitro* assays showed that MMP-8^−/−^ mice had lower Ang II and blood pressure levels than in controls. Consistent with these findings, the authors demonstrated that the plasma levels of Ang I, precursor of Ang II, were higher and blood pressure was lower in MMP8^−/−^ mice than those in the control group. These results are relevant since previous studies have demonstrated that Ang II induced the expression of adhesion molecules, such as VCAM-1, which has been shown to increase leukocyte intraplaque recruitment in atherogenesis [30, 31]. VCAM-1 plasma levels and its expression on ECs were substantially reduced in the MMP-8^−/−^ mice as compared to controls. As expected, in these knockout mice, the leukocyte rolling and adhesion on vascular endothelium were attenuated compared with those of controls [28]. This effect might be explained by the reduction of both Ang II and VCAM-1 levels in the MMP-8^−/−^ mice. Thus, MMP-8 might play a role in the development and progression of atherosclerosis also via degradation of nonmatrix proteins like Ang I. Since reduced blood pressure levels have been observed in MMP-8^−/−^ mice, an antiatherosclerotic mechanism independent of any other Ang II properties might be also considered. These mechanisms, as well as the identification of the sources of MMP-8 (vascular versus extravascular), merit further investigations.

## 3. Association of Circulating MMP-8 Levels and Atherosclerotic Disease

Given the low-grade systemic inflammation characterizing atherosclerosis, circulating mediators and acute phase proteins have been investigated together with intraplaque inflammation to better evaluate the global patient vulnerability [32, 33]. The most extensively studied serum biomarker in atherosclerosis is the C-reactive protein (CRP). Nevertheless, the predictive value of CRP as in atherosclerosis is only moderate [34]. It has been shown that some proteins derived from unstable plaques can be secreted into the bloodstream. For example, increased circulating levels of MMP-9 have been reported in patients with acute coronary syndromes, stable coronary artery disease, and carotid artery stenosis [35–37]. On the other hand, the circulating levels of this MMP correlate with the cardiovascular risk. The measurement of MMPs in the blood has been recommended as a noninvasive tool in diagnosis and monitoring [38]. Unfortunately, both serum and plasma MMP-8 concentrations have only rarely been determined in the context of cardiovascular diseases (CVD), so the possible association between circulating MMP-8 levels and the cardiovascular risk remains to be evaluated. Results from a cohort of patients undergoing carotid endarterectomy (*n* = 84) for symptomatic and asymptomatic disease have been recently published [39]. The authors demonstrated that MMP-8 plasma levels were significantly higher in patients with unstable, hypoechogenic plaques as assessed on ultrasound than those of subjects with stable, hyperechogenic plaques. Furthermore, plasma MMP-8 levels decreased during the time after ischemic stroke. Thus, the plasma MMP-8 concentrations have been found to be associated with carotid plaque instability, morphology, and the time after stroke. Another study was conducted in patients undergoing coronary angiography (*n* = 250) for coronary artery disease (CAD) [40]. Plasma MMP-8 concentrations were significantly higher in CAD patients as compared to those of subjects without the disease. Moreover, the authors observed a stepwise increase in MMP-8 concentration depending on the number of stenotic coronaries. In patients with unstable angina (UAP) (*n* = 45) and with stable CAD (*n* = 175), Momiyama and co-workers found that plasma MMP-8 levels were higher in patients with stable CAD than those of controls. They also noticed that MMP-8 levels in patients with UAP were much higher than those in stable CAD [41]. More recently, in two case-control studies (*n* = 141 and 343, resp.) high serum concentrations of MMP-8 and MMP-8/TIMP1 ratios were strongly associated with acute coronary syndrome [42, 43]. So, the results obtained in these “large” studies indicated that the plasma MMP-8 concentrations in patients with advanced atherosclerosis have been positively associated with the presence and severity of carotid artery plaque progression.

A decisive step was made by Tuomainen and coworkers in a prospective men-population-based (*n* = 1018) study with a clinical followup for 10 years [44]. Authors showed that serum MMP-8 concentration was an independent risk factor for acute myocardial infarction, CAD and CVD. The increased risk for CVD death was especially substantial in male patients with subclinical atherosclerosis at baseline. Indeed, high serum MMP-8 concentration increased by 3-fold the risk for CVD death during the followup independently of other known CVD risk factors. However, further analyses are required to confirm the clinical relevance of MMP-8 in CVD in a general population including women.

The present findings on circulating MMP-8 may have practical implications on both diagnosis and therapy of carotid and coronary diseases. Obviously, much more studies are needed before serum MMP-8 levels could be validated as a clinically useful marker for better evaluating the risk of acute cardiovascular events.

## 4. MMP-8 Gene Polymorphisms in Atherosclerosis

Considerable evidence has implied a role for MMPs in atherosclerosis. Nevertheless, evidence from expression studies and circulating markers cannot prove causality, because changes could be secondary to the disease phase. The study of polymorphisms, which are associated with lifelong changes in MMP activity, offers the possibility of determining whether such relationships are really causal. Using genetic variants in this way is referred as mendelian randomization and has been used to determine causality in cardiovascular diseases [45]. Several genetic association studies on the role of specific MMP variants in atherosclerosis have been performed, especially with MMP1, MMP-3, and MMP-9 [46–48]. These studies have demonstrated for example that the MMP-3 6A6A genotype is suggested to be associated with atherosclerosis and the 5A allele with plaque rupture. On the other hand, a meta-analysis of studies associating the MMP-9 polymorphisms with CAD found no evidence of an association [48].

The human MMP-8 gene is located on chromosome 11q22.2-q22.3 in a cluster of nine MMP genes and is composed of 12 exons [14, 49, 50]. Little is known about the functional properties of its promoter or about the transcriptional regulation of the gene, which is most likely a key regulator step, as has been observed for other MMPs. By sequencing the MMP-8 gene proximal promoter and coding regions and tagging single nucleotide polymorphisms (SNP) in the introns, 5′ upstream sequence and 3′-untranslated region in CAD patients (*n* = 1000), Laxton and colleagues have detected an association between the extent of coronary atherosclerosis and SNP rs19440475 [28]. Additional genotyping of CAD patients (*n* = 1000) for this SNP revealed a highly significant association between the SNP and extent of coronary atherosclerosis. Furthermore, the prospective study with this entire population (*n* = 2000) revealed that the T-allele of SNP rs1940475 was associated with a protective effect against carotid atherosclerosis progression in a 10-year followup. Interestingly, *in vitro *functional analysis indicated that the MMP-8 zymogen (the latent form of secreted MMP -8) with Lys87 (produced by the T allele) is less amenable to activation than the “native” zymogen with Glu87 (produced by the C allele) [28]. In summary, this genetic approach has demonstrated a significant relationship between a MMP-8 gene polymorphism and the progression of atherosclerosis.

More recently, Djurić and coworkers investigated the association of two promoter polymorphisms, rs1125395 (-799 C/T) and rs1320632 (-381 A/G), with plaque occurrence in Caucasians from Serbia (*n* = 766) [51]. These polymorphisms have been previously identified in African-Americans (*n* = 168), and *in vitro* data have demonstrated that these SNP confer increased MMP-8 promoter activity contributing to adverse events linked to extracellular matrix breakdown [52]. Interestingly, the presence of the -381 A/G polymorphism was not confirmed in studies of Asian populations, thus suggesting a diverse regulation of the promoter depending on the population analyzed [53, 54]. In their preliminary study, Djurić and coworkers showed a significant association between the -381G allele and the occurrence of carotid plaque in females [51]. However, further validation studies in a larger population are needed to verify the sex-specific association of this polymorphism with the presence of a carotid plaque. Additionally, significant higher expressions of MMP-8 mRNA were detected in atherosclerotic plaque tissue samples of carriers of the -381G allele and -381G/-799T haplotype. This result suggests that the MMP-8 promoter gene polymorphisms affect mRNA expression in human atherosclerotic plaque. Nevertheless, additional research is needed to further analyze functionality of the promoter polymorphisms and of the intraplaque mRNA-protein correlation before a conclusion can be drawn.

## 5. Conclusion

During the last decade, the studies presented here have provided novel and important insight into the role of MMP-8 in atherosclerosis. Indeed, due to its specific localization in immune cells and activity, MMP-8 is involved in the remodeling processes within the atherosclerotic plaque and promotes its rupture. In addition, the association between MMP-8 gene variation and atherosclerosis was validated in several population-based studies. Finally, only few studies demonstrated that MMP-8 concentrations (or MMP8/TIMP-1 ratios) may have prognostic and diagnostic significance in the assessment of patient's cardiovascular risk. Considering the multifactorial causes of atherosclerosis, it is probably too simplistic to assume that a single systemic or intraplaque biomarker (i.e., MMP-8) would suffice as a pathophysiological target for treatments and diagnosis. So, we believe that future studies might not only focus on MMP-8 alone but they have to consider this metalloproteinase as a promising parameter combined with a multimarker approach.
